# Inferences about moral character moderate the impact of consequences on blame and praise

**DOI:** 10.1016/j.cognition.2017.05.004

**Published:** 2017-10

**Authors:** Jenifer Z. Siegel, Molly J. Crockett, Raymond J. Dolan

**Affiliations:** a Department of Experimental Psychology, University of Oxford, United Kingdom; b Wellcome Trust Centre for Neuroimaging, University College London, United Kingdom; c Department of Psychology, Yale University, USA; ^d^ Max Planck Centre for Computational Psychiatry and Ageing, University College London, United Kingdom

**Keywords:** Morality, Moral judgment, Omission bias, Blame, Praise, Character

## Abstract

Moral psychology research has highlighted several factors critical for evaluating the morality of another’s choice, including the detection of norm-violating outcomes, the extent to which an agent caused an outcome, and the extent to which the agent intended good or bad consequences, as inferred from observing their decisions. However, person-centered accounts of moral judgment suggest that a motivation to infer the moral character of others can itself impact on an evaluation of their choices. Building on this person-centered account, we examine whether inferences about agents’ moral character shape the sensitivity of moral judgments to the *consequences* of agents’ choices, and agents’ role in the *causation* of those consequences. Participants observed and judged sequences of decisions made by agents who were either bad or good, where each decision entailed a trade-off between personal profit and pain for an anonymous victim. Across trials we manipulated the magnitude of profit and pain resulting from the agent’s decision (consequences), and whether the outcome was caused via action or inaction (causation). Consistent with previous findings, we found that moral judgments were sensitive to consequences and causation. Furthermore, we show that the inferred character of an agent moderated the extent to which people were sensitive to consequences in their moral judgments. Specifically, participants were more sensitive to the magnitude of consequences in judgments of bad agents’ choices relative to good agents’ choices. We discuss and interpret these findings within a theoretical framework that views moral judgment as a dynamic process at the intersection of attention and social cognition.

## Highlights

• We studied how inferences about moral character affect blame and praise judgments.
• Blame and praise judgments were sensitive to character, consequences and causation.
• Inferring bad character amplified effects of consequences on judgments.

## 1 Introduction

A longstanding question in moral psychology is a concern with the criteria people use when assigning blame to others’ actions. Theories of blame highlight several critical factors in determining an agent’s blameworthiness for a bad outcome (Alicke et al., 2015, Heider, 1958, Malle et al., 2014, Shaver, 1985, Weiner, 1995). The first step is detecting some bad outcome that violates a social norm. Next comes an evaluation of whether the agent caused the outcome, followed by an assessment of whether the agent intended the outcome. People are considered more blameworthy for harmful actions than equally harmful omissions (Baron, 1994, Baron and Ritov, 1994, Cushman et al., 2012, Spranca et al., 1991) because the former are viewed as more causal than the latter (Cushman & Young, 2011). Moreover, people are blamed more for intentional compared to unintentional (i.e., accidental) harms (Karlovac and Darley, 1988, Shultz and Wright, 1985, Shultz et al., 1986). Causation and malintent are each alone sufficient to ascribe judgments of blame for bad outcomes. In the case of accidental harms, people blame agents for bad outcomes that they caused but did not intend (Ahram et al., 2015, Cushman, 2008, Cushman et al., 2009, Martin and Cushman, 2015, Oswald et al., 2005). There is also evidence that people blame agents for bad outcomes that they intend or desire but do not cause (Cushman, 2008, Inbar et al., 2012).

Other work has highlighted how inferences about moral character impact the assignment of blame and praise. For example, judges and juries frequently condemn repeat offenders to harsher penalties than first-time offenders for equivalent crimes (Roberts, 1997), and conviction rates are correlated with jurors’ knowledge of a defendant’s previous crimes (T. Eisenberg & Hans, 2009), particularly when past crimes are similar to a current offence (Alicke et al., 2015, Wissler and Saks, 1985). In the laboratory, people assign more blame to dislikable agents than likable agents (Alicke and Zell, 2009, Kliemann et al., 2008, Nadler, 2012). These observations are consistent with a *person-centered* approach to moral judgment, which posits that evaluations of a person’s moral character bleed into evaluations of that person’s actions (Uhlmann, Pizarro, & Diermeier, 2015). In other words, despite being instructed to assess whether an *act* is blameworthy, people may instead evaluate whether the *person* is blameworthy.

In line with this view, there is evidence that evaluations of causation and intent are themselves sensitive to inferences about an agent’s character (Alicke, 1992, Alicke, 2000, Knobe, 2010, Knobe and Fraser, 2008, Mazzocco et al., 2004). That is, people tend to conflate moral evaluations of agents with their perceptions of agents’ intentions and causation. For example, in the culpable control model of blame, a desire to assign blame to disliked agents influences perceptions of their control over an accident (Alicke, 2000; but see Malle et al., 2014). In an early demonstration of this phenomenon, participants were told that a man speeding home got into a car accident, leaving another person severely injured (Alicke, 1992). The man was described as rushing home to hide either an anniversary present or a vial of cocaine from his parents. Participants judged the delinquent cocaine-hiding individual as having more control by comparison to the virtuous present-hiding man. Similar effects are seen when participants are given more general information about the agent’s character (Alicke and Zell, 2009, Nadler, 2012). People also judge an agent who breaks a rule as being more causally responsible for an outcome that breaks a rule than an agent who takes the same action but does not break a rule, suggesting negative moral evaluations increase causal attributions (Hitchcock & Knobe, 2009).

Moral judgments of agents also affect evaluations of intent. For instance, harmful foreseen side-effects are seen as more intentional than helpful foreseen side effects, suggesting that negative moral evaluations lower the threshold for inferring intentionality (Alicke, 2008, Knobe, 2010, Knobe and Fraser, 2008, Nadelhoffer, 2004, Ngo et al., 2015, Uhlmann et al., 2015). In a study where participants played an economic game with agents who were either trustworthy or untrustworthy, and then evaluated the extent to which the agents intended various positive and negative outcomes, the untrustworthy agent was more likely to be evaluated as intending negative outcomes than the trustworthy agent (Kliemann et al., 2008). Greater activation was seen in the right temporoparietal junction, a region implicated in evaluating intent, when assigning blame to an untrustworthy relative to a trustworthy agent (Kliemann et al., 2008). Thus there is a substantial literature supporting a ‘person-as-moralist’ view of blame attribution (Alicke et al., 2015, Knobe, 2010, Tetlock, 2002), which posits that people are fundamentally motivated to assess the goodness and badness of others, and perceive others’ intent and causation in a way that is consistent with their moral evaluations.

To assign blame and praise it is necessary to infer an agent’s mental state based on their actions, by considering the likely end consequences of their action (Malle, 2011). Recent work has shown that from an early age people readily infer people’s intentions by observing their decisions, deploying a “naïve utility calculus” that assumes people’s choices are aimed at maximizing desirable consequences and minimizing undesirable consequences, where desirability is evaluated with respect to the agent’s preferences (Jara-Ettinger, Gweon, Schulz, & Tenenbaum, 2016). This means that in situations where agents make deterministic choices, their intentions can be inferred from the consequences of their choices. Evaluations of moral character are intimately linked to inferences about intentions, where accumulated evidence of bad intent leads to a judgment of bad character (Leifer, 1971, Leslie et al., 2006, Uhlmann et al., 2015). What remains unknown is whether, and how, the formation of character beliefs impacts on moral judgments of individual actions. In other words, when people repeatedly observe an agent bring about either harmful or helpful consequences, do learnt inferences about the agent’s character influence how people make judgments regarding the agent’s individual acts?

**Fig. 1:**
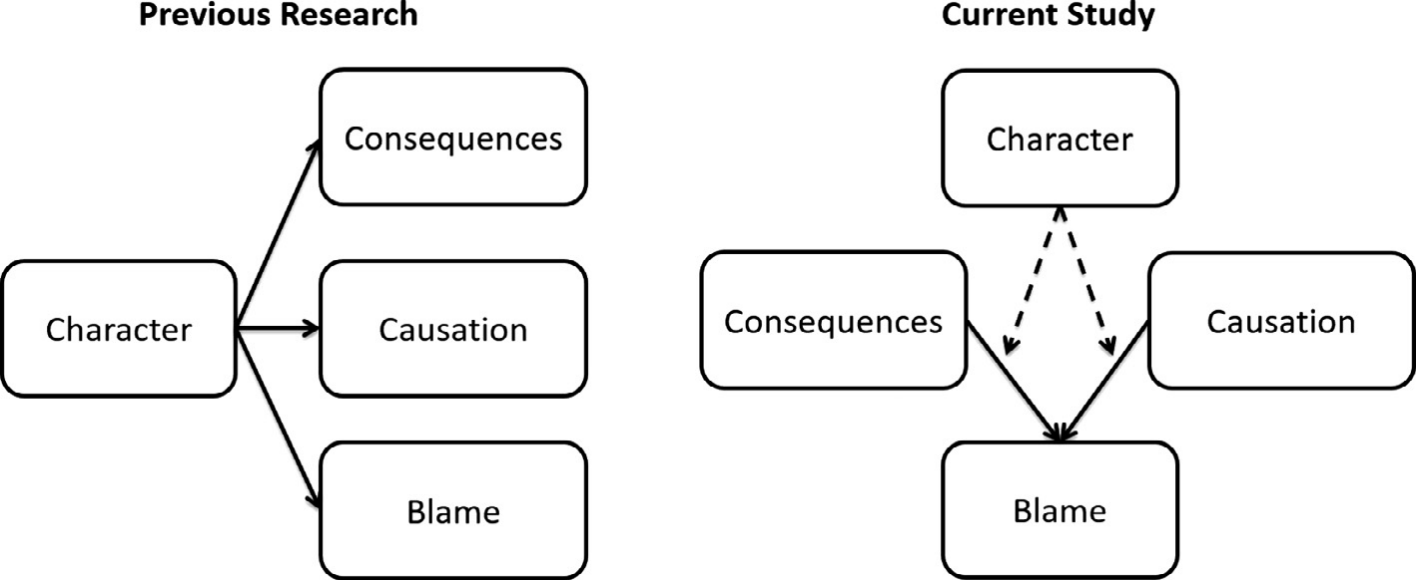
Previous work has investigated the effects of character perception on separate explicit (i.e., self-reported) judgments of consequences, causation, and blame. In the current study we investigate how character perception *moderates* the impact of consequences and causation on blame judgments. Consequences and causation were manipulated within the task structure.

Our research addresses several open questions. First, although studies have shown that perceptions of character influence separate assessments of consequences, causation, and blameworthiness, it remains unknown how precisely character evaluations affect the *degree to which* consequences and causation shape blame attributions (Fig. 1). Second, the bulk of research in this area has focused on judgments of blameworthiness for harmful actions with less attention to how people judge praiseworthiness for helpful actions (Cushman et al., 2009, Pizarro et al., 2003, Weiner, 1995). Furthermore, those studies that have investigated praiseworthy actions have generally used scenarios that differ from those used in studies of blame not only in terms of their moral status but also in terms of their typicality. For example, studies of blame typically assess violent and/or criminal acts such as assault, theft, and murder, while studies of praise typically assess good deeds such as donating to charity, giving away possessions or helping others with daily tasks (Eisenberg et al., 2001, Pizarro et al., 2003). Thus, our understanding of how consequences and causation impact judgments of blame versus praise, and their potential moderation by character assessments, is limited by the fact that previous studies of blame and praise are not easily comparable.

In the current study we used a novel task to explore how inferences about moral character influence the impact of consequences and causation on judgments of blame and praise for harmful and helpful actions. Participants evaluated the blameworthiness or praiseworthiness of several agents’ harmful or helpful actions. These varied across trials, in terms of their *consequences* and also in terms of the degree to which the actions *caused* a better or worse outcome for a victim. In Study 1, participants evaluated a total of four agents: two with *good character,* and two with *bad character.* In Study 2 we replicate the effects of Study 1 in a truncated task where participants evaluated one agent with good character and one agent with bad character. We used linear mixed models to assess the extent to which blame and praise judgments were sensitive to the agents’ consequences, the agents’ causation of the outcomes, the agents’ character, and the interactions among these factors. The advantage of this approach is that it allows us to capture the influence of consequences, causation, and character on integrated moral judgments, without requiring participants to directly report their explicit (i.e., self-reported) evaluations of these cognitive subcomponents (Crockett, 2016) (Fig. 1). For example, we can measure whether the effects of perceived causation on blame differs for good and bad agents, without asking participants directly about the perceived causation of good vs. bad agents. With this approach we can more closely approximate the way assessments of consequences and causation influence blame judgments in everyday life, where people might assign blame using implicit, rather than explicit, evaluations of causation and consequences.

**Fig. 2:**
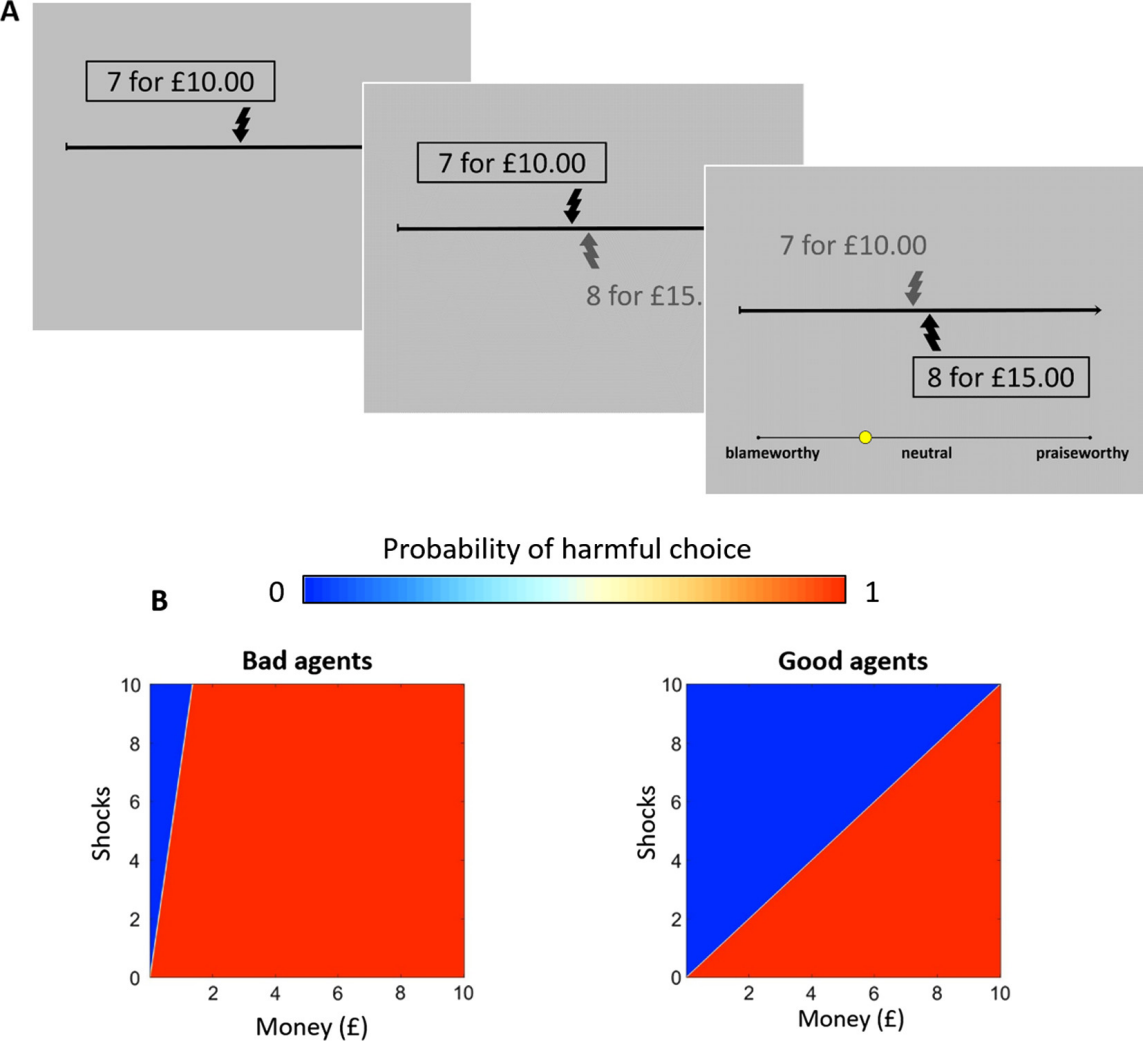
Trial structure for the moral judgment task. (A) Each trial consisted of three screens that began by indicating a default number of shocks and money an agent would receive if that agent *did nothing* (above). This was followed by an alternative number of shocks and money that the agent would receive if they decided to *switch* (below)*.* Finally, the agent’s choice was revealed and participants judged each choice on a scale ranging from ‘blameworthy’ to ‘praiseworthy’. (B) Heat maps depicting the bad and good agents’ probability of choosing the more harmful option as a function of the money gained and shocks delivered. As the option becomes more profitable (i.e., as more money is offered at the cost of each shock), the probability of choosing the harmful option increases, but the bad agents require less profit to choose the harmful option than do the good agents.

We manipulated the agents’ consequences by having the agents choose, on each trial, between a *harmful option* that yields a higher monetary reward at the expense of delivering a larger number of painful electric shocks to an anonymous victim, and a *helpful option* that yields a lower monetary reward but results in fewer painful shocks delivered to the victim (Fig. 2A). Across trials we varied the amount of profit and pain that result from the harmful relative to the helpful option. Thus, an agent in choosing the harmful option might inflict a small or large amount of pain on the victim for a small or large profit. Likewise, for helpful actions, an agent might sacrifice a small or large amount of money to reduce the victim’s pain by a small or large amount. We predicted that participants would infer the agents’ intentions from their choices and assign blame and praise accordingly: an agent who is willing to inflict a given amount of pain for a small profit should be blamed more than an agent who is only willing to inflict the same amount of pain for a much larger profit. Likewise, an agent who is willing to sacrifice a large amount of money to reduce pain by a given amount should be evaluated as more praiseworthy than an agent who is only willing to sacrifice less money to achieve the same benefit. Such evaluations would be consistent with the idea that people infer the intentions of others according to a “naïve utility calculus” where agents choose so as to minimize costs and maximize rewards (Jara-Ettinger et al., 2016).

We manipulated causation by having the agents cause the harmful and helpful outcomes either via an overt action, or via inaction. Previous work has shown that the well-documented ‘omission bias’, whereby harm brought about by an action is judged worse than harm brought about by a failure to act (Baron and Ritov, 1994, Kordes-de Vaal, 1996, Spranca et al., 1991), can be explained primarily by causal attribution (Cushman & Young, 2011). That is, an agent who brings about harm via an action is seen as *causing* the harm more than an agent who brings about harm by failing to act. At the start of each trial, a default option was highlighted and the agent could switch from the default to the alternative by pressing a key within a time limit. On half the trials the agent switched, while on the other half the agent did nothing. Crucially, across trials we matched the amount of help and harm that resulted from switching versus doing nothing, so that the agents brought about identical outcomes both via action and inaction. We predicted that participants would assign more blame for the same harmful outcomes brought about via action than inaction, and that they would assign more praise for the same helpful outcomes brought about via action than inaction, consistent with previous studies (Baron, 1994, Baron and Ritov, 1994, Cushman et al., 2012, Johnson and Drobny, 1987, Spranca et al., 1991).

Finally, we manipulated character by having agents choose according to different exchange rates for money and pain: *good agents* required a high profit to inflict pain on others (£2.43 per shock), and were willing to sacrifice large amounts of money to reduce a victim’s pain; *bad agents* required only a small profit to inflict pain (£0.40 per shock) and were only willing to sacrifice small amounts of money to reduce a victim’s pain (Fig. 2B). Previous studies investigating how people actually make choices in this setting demonstrated the amount of money people are willing to trade for others’ pain correlates with morally relevant traits, including empathy and psychopathy (Crockett, Kurth-Nelson, Siegel, Dayan, & Dolan, 2014). Although good and bad agents by definition made different choices, on a subset of trials they made the same choice. Consistent with studies showing people assign more blame to disliked individuals (Alicke and Zell, 2009, Kliemann et al., 2008, Nadler, 2012), we predicted that bad agents would be blamed more than good agents, even when making identical choices.

## 2 Methods

### 2.1 General procedure

Two studies were conducted at the Wellcome Trust Centre for Neuroimaging in London, UK and were approved by University College London (UCL) Research Ethics Committee (4418/001). Participants in both studies completed a battery of trait questionnaires online prior to attending a single testing session. Each session included two participants who were led to separate testing rooms without seeing one another to ensure complete anonymity. After providing informed consent, a titration procedure was used to familiarize participants with the electric shock stimuli that would be used in the experiment. Subjects were then randomly assigned to one of two roles: the ‘decider’ who engaged in a moral decision task, or the ‘receiver’ who completed a moral judgment task. In Study 1, participants assigned to the role of the receiver completed the moral judgment task. In Study 2, participants assigned to the role of the decider in an entirely separate sample (i.e., not paired with the receivers from Study 1) completed the moral judgment task after completing the moral decision task. Here, we focus on behavior in the moral judgment task alone. Data from the moral decision task in Study 2 is reported elsewhere (Crockett et al., 2014).

### 2.2 Participants

Healthy volunteers (Study 1: N = 40, 16 men; Study 2, N = 40, 14 men) were recruited from the UCL psychology department and the Institute of Cognitive Neuroscience participant pools. All participants provided written informed consent prior to participation and were financially compensated for their time. Participants with a history of systemic or neurological disorders, psychiatric disorders, medication/drug use, pregnant women, and more than two years’ study of psychology were excluded from participation. Furthermore, to minimize variability in participants’ experiences with the experimental stimuli, we excluded participants previously enrolled in studies involving electric shocks. Power calculations indicated that to detect effects of moderate size (*d* = 0.5) with 80% power, we required a sample of at least 34 participants. The current samples were thus adequately powered to detect moderate effects of our experimental manipulations.

### 2.3 Experimental design

As previously stated, participants entered the laboratory in pairs and were then randomized into the role of either ‘decider’ or ‘receiver’. Both participants were then informed of the decider’s task (the moral decision task), which involved choosing between delivering more painful electric shocks for a larger profit, and delivering fewer shocks but for a smaller profit. For each trial of the moral decision task, there was a default option and an alternative. The default option would automatically be implemented if the decider did nothing, but deciders could switch from the default to the alternative by making an active response. The decider alone received money from their decisions, but shocks were sometimes allocated to the decider and sometimes allocated to the receiver. Participants were informed that at the end of the decider’s task, one of the decider’s choices would be randomly selected and implemented. Thus, participants assigned to the role of the receiver (participants in Study 1) were aware that they could receive harmful outcomes (electric shocks) resulting from the decisions of another person. Conversely, participants assigned to the role of the decider (participants in Study 2) were aware that their decisions could result in a degree of harm to another person.

In Study 1, participants completed a moral judgment task in which they evaluated sequences of 30–32 choices made by four fictional deciders (here, called “agents”), presented one at a time in random order, for a total of 124 trials. After observing a given choice, participants provided a moral judgment of the choice on a continuous visual analogue scale ranging from 0 (*blameworthy*) to 1 (*praiseworthy*) (Fig. 2**A**). In Study 2, participants completed a similar task where they evaluated sequences of 30 choices made by two agents, presented one at a time in random order, for a total of 60 trials. Participants in Study 1 were instructed that the agents whose choices they were evaluating reflected the choices of previous deciders and were not the choices of the current decider in the next room. Participants in Study 2 were instructed that the agents whose choices they were evaluating reflected the choices of previous deciders. For full instructions and trial parameters, see Supplementary Materials).

Across trials for a given agent we manipulated the following factors:

• *Consequences:* the difference in the number of shocks and amount of money that resulted from the agent’s choice. These numbers could be negative (helpful, costly choices for the agent) or positive (harmful, profitable choices for the agent). The difference in number of shocks ranged from −9 to 9, while the difference in amount of money ranged from -£9.90 to £9.90. Thus, in Fig. 2a, the difference in shocks was equal to 1 shock and the difference in money was equal to £5.00. The precise amounts of shocks and money resulting from harmful and helpful choices were sufficiently de-correlated across trials (correlation coefficients <0.7, Dormann et al., 2013; Supplementary Table 1) enabling us to examine independent effects of shocks and money on judgments in our regression analysis. Additionally, this manipulation enabled a parametric analysis examining harmfulness and profit on a continuous scale.
• *Causation:* on half the trials, agents chose to switch from the default to the alternative option (action trials). On the other half, agents chose to stick with the default option (inaction trials). Action and inaction trials were matched in terms of consequences so we could directly compare judgments of harmful actions with equally harmful inactions, and helpful actions with equally helpful inactions. Because actions are perceived as more causal than inactions (Cushman & Young, 2011), this manipulation enabled us to investigate the extent to which moral judgments are sensitive to differences in the agents’ *causal role* for bringing about the outcome. Across trials, the default number of shocks varied from 1 to 20, while the default amount of money was always £10.00.
• *Character:* To investigate how character influences judgments we manipulated the moral preferences of the agents. Specifically, each agent’s moral preferences were determined by a computational model of moral decision-making validated in previous experiments (Crockett et al., 2014, Crockett et al., 2015, Fig. 2**B**). In this model, the subjective cost of harming another is quantified by a harm aversion parameter, κ. When ln(κ) → −∞, agents are minimally harm-averse and will accept any number of shocks to increase their profits; as ln(κ) → ∞, agents become increasingly harm-averse and will pay increasing amounts of money to avoid a single shock.

Participants in Study 1 judged the choices of four agents: two bad agents (agents B1 and B2; ln(κ) = −2.) and two good agents (agents G1 and G2; ln(κ) = 0). The agents’ choices were determined by a computational model that described the value of switching from the default to the alternative (V_act_) as a function of the difference in money, Δm, and difference in shocks, Δs, scaled by κ for agent *i*:

(1) $${\text{V}}_{\text{act}}=\Delta \text{m}-\Delta \text{s}*e^{\text{ln}(\kappa_{i})}$$

Agents B1 and G1 faced identical choice sets but made different proportions of harmful vs. helpful choices, with agent B1 harming on 66% of trials and agent G1 harming on 33% of trials. These two agents made identical choices 66% of the time, which allowed us to compare subjects’ judgments on trials where the bad and good agent made identical choices. Agents B2 and G2 faced choice sets with different incentives that induced each to choose the more harmful option on 50% of trials. This permitted us to compare subjects’ judgments of choices resulting in equivalent shocks, but for different amounts of money; the good agent required a greater profit for equivalent increases in shocks, and would accept greater losses for equivalent decreases in shocks, relative to the bad agent.

Participants in Study 2 only evaluated the choices of agents B1 and G1 after completing the moral decision task. This allowed us to focus our analysis on choices where agents faced identical choice sets and behaved similarly most of the time. In both studies, three sequences of trials were generated and randomized across participants. See Supplemental Materials for details about agent simulations.

Also in both studies, after observing the full sequence of choices for each agent, participants rated two aspects of the agent’s character (kindness and trustworthiness) and three aspects of the agent’s choices (harmfulness, helpfulness, selfishness). Each rating was provided on a continuous visual analogue scale ranging from 0 (*not at all*) to 1 (*extremely*). The exact wordings of the questions were as follows:

*Kindness:* “In your opinion, how KIND was this Decider?”
  *Trustworthiness:* “How much would you TRUST this Decider?”
  *Harmfulness:* “Please consider all of the Decider’s choices. In your opinion, what proportion of the Decider’s choices were HARMFUL?”
  *Helpfulness:* “Please consider all of the Decider’s choices. In your opinion, what proportion of the Decider’s choices were HELPFUL?”
  *Selfishness:* “Please consider all of the Decider’s choices. In your opinion, how SELFISH was this Decider?”

### 2.4 Analysis

We analysed the data using a number of complementary approaches. In a regression analysis we modelled participants’ trial-by-trial moral judgments of all agents in a linear mixed-effects model with random intercepts and slopes. The regressors in the model included: moral character (‘character’, *β*_1_), the absolute difference between the chosen and unchosen amounts of shocks (‘shocks’, *β*_2_), the absolute difference between the chosen and unchosen amounts of money (‘money’, *β*_3_), causation of consequences (dummy coding for action vs. inaction, *β*_4_), the interaction between character and shocks (*β*_5_), the interaction between character and money (*β*_6_), and the interaction between character and causation (*β*_7_). Character was operationalized as a categorical regressor describing the effect of *good* agents on moral judgments. Our regression included an intercept term, *c,* capturing the average judgment across trials, and all other coefficients expressed mean deviations from this judgment.

(2) $$\mathrm{Judgment}=\beta_{1}\left(\kappa \right)+\beta_{2}\left(\Delta s\right)+\beta_{3}\left(\Delta m\right)+\beta_{4}\left(\mathrm{causation}\right)+\beta_{5}\left(\Delta s*\kappa \right)+\beta_{6}\left(\Delta m*\kappa \right)+\beta_{7}\left(\mathrm{causation}*\kappa \right)+c$$

We focused on the absolute magnitude of each weight, rather than its directionality, because this provided us with estimates of how sensitive people were to the different features of the choice. In analysing the independent effects of character, consequences, and causation, we aimed to validate our approach by replicating findings previously reported using scenario-based methods, such as increased blame for more harmful consequences, and increased blame for harmful actions relative to inactions. Subsequent analyses of the interaction terms allowed us to investigate the effects of moral character on sensitivity to each main effect.

Our primary analysis used a categorical character regressor in the model. However, to verify our results we also fit the model described in Eq. (2) substituting the categorical ‘character’ regressor with participants’ own subjective ratings of the agents’ kindness. We chose to focus specifically on the kindness character rating because our task was not designed to measure trust.

We fit the data using a linear mixed-effects model with random intercepts in R (lmerTest package). Estimates of fixed effects are reported along with standard error (SE, *M* = mean ± SE).

In a subsequent exploratory analysis, we examined the independent influence of moral character on: (a) how sensitive people were to consequences in attributions of blame, and (b) how sensitive people were to consequences in attributions of praise. To this end, we fit each parameter in our linear model separately for harmful trials (where the agent chose the option with more shocks) and helpful trials (where the agent chose the option with fewer shocks). Because we did not find a significant interaction between character and causation in our previous analysis, we omitted these regressors from the model.

(3) $$\begin{array}{cc} & \mathrm{Judgment}=\beta_{1}\left(\kappa \right)+\beta_{2}\left(\Delta s\right)+\beta_{3}\left(\Delta m\right)+\beta_{4}\left(\mathrm{causation}\right)+\beta_{5}\left(\Delta s*\kappa \right)+\beta_{6}\left(\Delta m*\kappa \right)+c\\ & \beta_{2}\text{,}\beta_{3\text{,}}\beta_{4}\text{,}\beta_{5}\text{,}\beta_{6}=\left\{\begin{array}{cc} \beta_{2+}\text{,}\beta_{3+}\text{,}\beta_{4+}\text{,}\beta_{5+}\text{,}\beta_{6+} & \text{if help trial}\\ \beta_{2-}\text{,}\beta_{3-}\text{,}\beta_{4-}\text{,}\beta_{5-}\text{,}\beta_{6-} & \text{if harm trial} \end{array}\right) \end{array}$$

We modelled participants’ moral judgments of all agents using the same linear mixed effects procedure in R.

Where possible, we confirmed the findings of our linear mixed-effects models with analyses that did not rely on a model. To do this we computed mean judgments for each cell of our 2 (harmful vs. helpful) × 2 (action vs. inaction) × 2 (good vs. bad agent) design on the subset of trials where good and bad agents made identical choices (Supplementary Table S2). We entered these mean judgments to a repeated-measures analysis of variance (ANOVA) and compared the results of this analysis to the results from our model.

## 3 Results

### 3.1 Manipulation checks

Participants’ post hoc ratings of the agents suggested they accurately inferred the agents’ moral character from the choices they made. Relative to bad agents, participants rated good agents’ character as significantly more kind (Study 1, Bad: *M* = 0.331 ± 0.024; Good = 0.732 ± 0.020; *t* = −14.326, p < 0.001; Study 2, Bad: *M* = 0.453 ± 0.032; Good = 0.749 ± 0.024; *t* = −9.072, p < 0.001) and trustworthy (Study 1, Bad: *M* = 0.322  ± 0.023; Good = 0.686 ± 0.023; *t* = −11.655, p < 0.001; Study 2, Bad: *M* = 0.463 ± 0.034; Good = 0.735 ± 0.030; *t* = −8.045, p < 0.001). Participants also rated good agents’ choices as more helpful (Study 1, Bad: *M* = 0.406  ± 0.025; Good = 0.687 ± 0.022; *t* = −9.613 p < 0.001; Study 2, Bad: *M* = 0.435 ± 0.032; Good = 0.722 ± 0.029; *t* = −10.597, p < 0.001), less harmful (Study 1, Bad: *M* = 0.641  ± 0.022; Good = 0.325 ± 0.025; *t* = 10.663, p < 0.001; Study 2, Bad: *M* = 0.606 ± 0.037; Good = 0.382 ± 0.041; *t* = 6.542, p < 0.001) and less selfish (Study 1, Bad: *M* = 0.657  ± 0.026; Good = 0.328 ± 0.025; *t* = 9.375, p < 0.001; Study 2, Bad: *M* = 0.635 ± 0.028; Good = 0.353 ± 0.032; *t* = 8.568, p < 0.001) than bad agents’ choices.

**Fig. 3:**
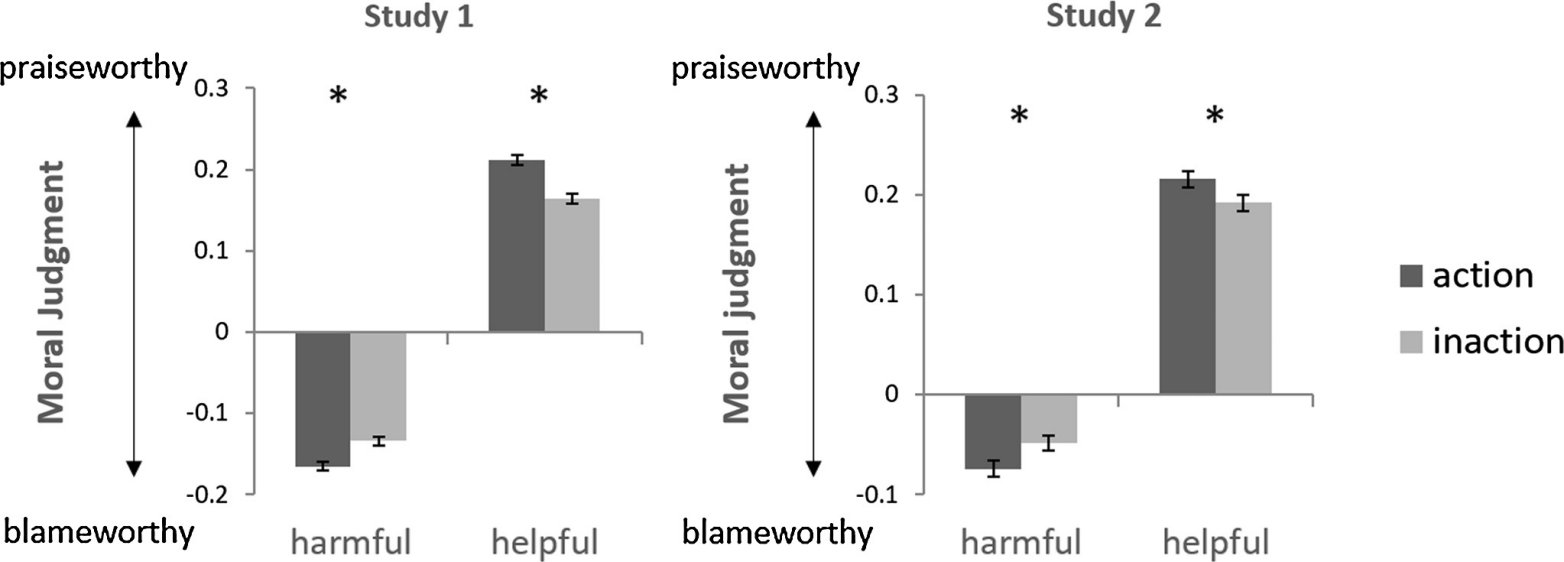
Causation moderates moral judgments of harmful and helpful behavior. Across all agents, harmful actions were more blameworthy than harmful inactions, and helpful actions were more praiseworthy than helpful inactions. Y-axis, represents the mean judgment (from 0 = *blameworthy* to 1 = *praiseworthy*) subtracted by the midpoint of the scale (0.5 = *neutral*). Error bars represent standard error of the difference between means.

Next we asked whether our within-task manipulations of consequences and causation exerted significant effects on moral judgments. To do this, we computed across all agents and trials the average judgments for each cell of our 2 (harmful vs. helpful) × 2 (action vs. inaction) × 2 (good vs. bad agent) design and subjected these mean judgments to a repeated-measures ANOVA. As expected, there was a significant effect of consequences on moral judgments [Study 1: F(1, 39) = 551.879, p < 0.001; Study 2: F(1, 39) = 70.385, p < 0.001], indicating that harmful choices were judged more blameworthy than helpful choices. As can be seen in Fig. 3a-b, judgments of helpful choices were above the midpoint of the scale and harmful choices were below the midpoint of the scale. This suggests that participants did in fact believe that helpful actions were deserving of praise, despite the fact that all helpful choices resulted in some degree of harm.

The main effect of causation was significant in Study 1 [F(1, 39) = 4.651, p = 0.037], though not in Study 2 [F(1, 39) = 0.040, p = 0.843], indicating actions were judged as more praiseworthy than inactions for Study 1 alone. This was qualified by a statistically significant interaction between causation and consequences on moral judgments in both studies [Study 1, F(1, 39) = 73.068, p < 0.001; Study 2, F(1, 39) = 22.121, p < 0.001; Fig. 3a and b]. Simple effects analyses showed that participants judged harmful actions as more blameworthy than harmful inactions (Study 1, *t* = −5.589, p < 0.001; Study 2, *t* = −3.222, p = 0.003), and helpful actions as more praiseworthy than helpful inactions (Study 1, *t* = 7.479, p < 0.001; Study 2, *t* = 2.869, p = 0.007). This analysis verified that within our design, moral judgments were strongly influenced both by the consequences of actions and by the causal role the agents played in producing the consequences.

### 3.2 Main effects of character on moral judgment

**Fig. 4:**
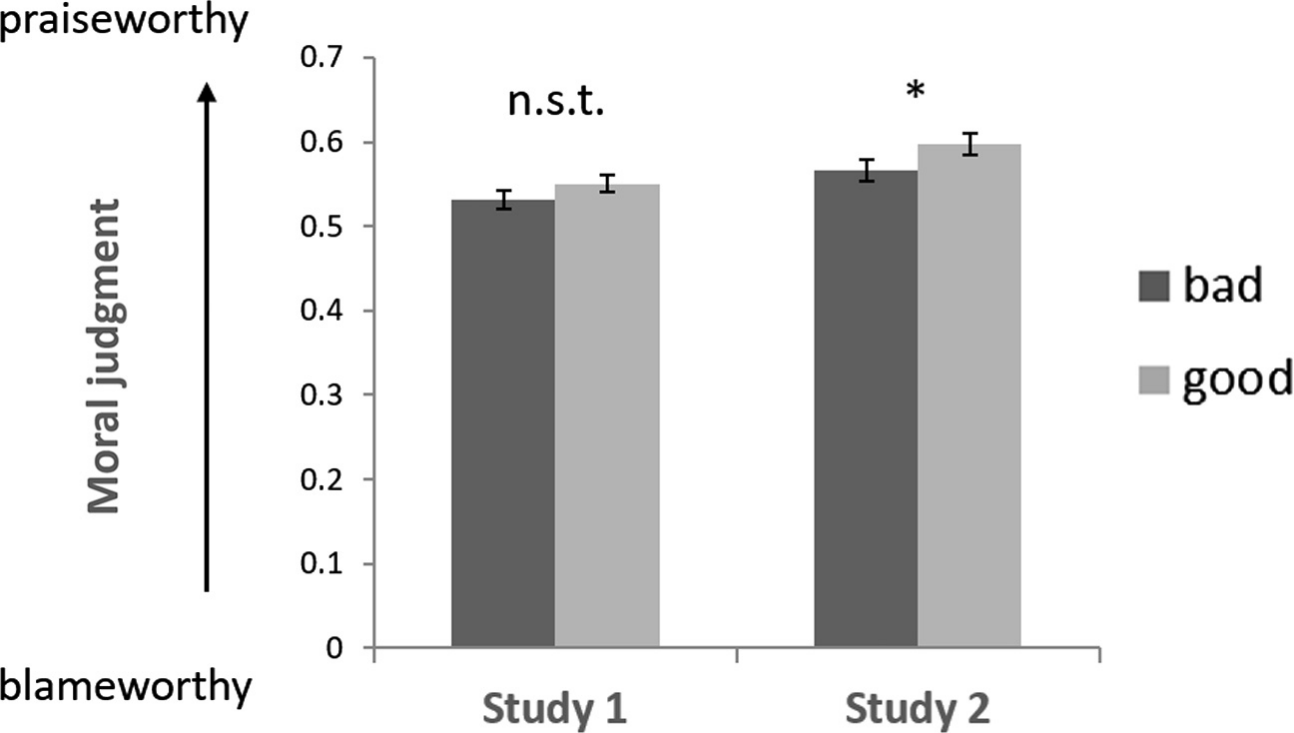
Overall effect of character on moral judgment. Across trials where good and bad agents behave identically, bad agents’ choices are evaluated more harshly than good agents’ choices. Error bars represent standard error of the difference between means. ^*^P < 0.05; n.s.t. = non-significant trend.

Next we examined the estimates from our model (Eq. (2)), which showed that controlling for all other factors, there was a small but significant effect of moral character on judgment. As predicted, bad agents were ascribed more blame than good agents (Study 1, *β*_1_ = 0.077 ± 0.006, *t* = 12.112, p < 0.001; Study 2, *β*_1_ = 0.093 ± 0.016, *t* = 5.658, p < 0.001). We repeated our analysis substituting the categorical ‘character’ regressor with participants’ own subjective ratings of the agents’ kindness and obtained the same results (Study 1, *β*_1_ = 0.178 ± 0.012, *t* = 15.330, p < 0.001; Study 2, *β*_1_ = 0.161 ± 0.031 *t* = 5.234, p < 0.001; See Supplementary Materials for full model results). Our model results were consistent with a complementary analysis in which we computed mean judgments for each cell of our 2 (harmful vs. helpful) × 2 (action vs. inaction) × 2 (good vs. bad agent) design on the subset of trials where good and bad agents made identical choices. Here, we observed a trend towards more favourable judgments of good than bad agents in Study 1 [F(1, 39) = 3.314, p = 0.076], and significantly more favourable judgments of good than bad agents in Study 2 [F(1, 39) = 5.774, p = 0.021]. Thus, for the *exact same* choices, bad agents received slightly harsher judgments than good agents, Fig. 4.

### 3.3 Character moderates the effects of consequences on moral judgments

**Fig. 5:**
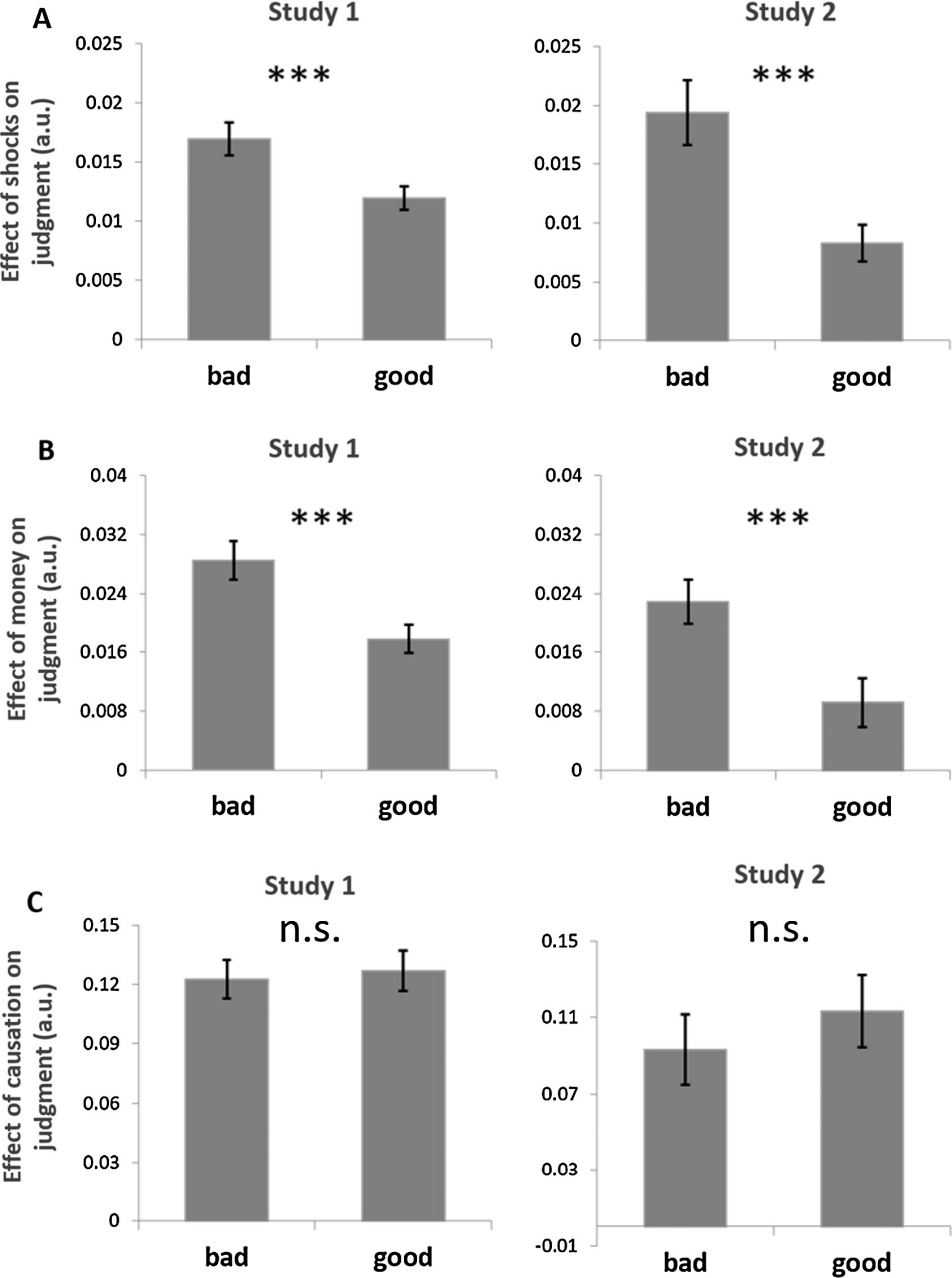
Character moderates the effects of consequences on moral judgment. Judgments of bad agents’ choices were more sensitive to the magnitude of shocks (A) and the magnitude of money (B) than judgments of good agents’ choices. Judgments of good and bad agents’ choices were similarly sensitive to the causal role the agents had in bringing about the consequences. Error bars represent standard error of the mean. Figures illustrate which interactions were statistically significant in Eq. (2). Y-axis represents the magnitude of the effect on moral judgment. A.u. = arbitrary units, ^***^P < 0.001; n.s. = not significant.

Parameter estimates for shocks, money, and causation in Eq. (2) were all significantly different from 0, indicating that moral judgments were independently affected by the number of shocks delivered to the victim (Study 1, *β*_2_ = 0.016 ± 0.001, *t* = 13.240, p < 0.001; Study 2, *β*_2_ = 0.019 ± 0.002, *t* = 8.815, p < 0.001), the amount of money received by the agent (Study 1, *β*_3_ = 0.028 ± 0.001, *t* = 23.707, p < 0.001; Study 2, *β*_3_ = 0.023 ± 0.002, *t* = 12.333, p < 0.001), and whether the agent made an active or passive choice (Study 1, *β*_4_ = 0.127 ± 0.007, *t* = 17.708, p < 0.001; Study 2, *β*_4_ = 0.097 ± 0.013, *t* = 7.373, p < 0.001). Furthermore, moral character moderated participants’ sensitivity to consequences. The interaction between character and shocks was significantly negative in both studies (Study 1, *β*_5_ = −0.004 ± 0.002, *t* = −2.535, p = 0.011; Study 2, *β*_5_ = −0.010 ± 0.003, *t* = −3.166, p = 0.002). The interaction between character and money was also significantly negative in both studies (Study 1, *β*_6_ = −0.010 ± 0.002, *t* = −6.787, p < 0.001; Study 2, *β*_6_ = −0.015 ± 0.004, *t* = −4.214, p < 0.001). Negative parameter estimates indicate that judgments of bad agents’ choices were significantly more sensitive to consequences than judgments of good agents’ choices. Meanwhile judgments of bad and good agents’ choices did not differ in terms of their sensitivity to causation (Study 1, *β*_7_ = 0.002 ± 0.010, *t* = 0.177, p = 0.860; Study 2, *β*_7_ = −0.0.008 ± 0.018, *t* = 0.446, p = 0.656). To illustrate these interaction effects, we estimated the shocks, money and causation parameters separately for the good and bad agents and display these in Fig. 5a–c.

### 3.4 Effects of character and consequences on blame vs. praise

In an exploratory analysis we modelled the effects of character, consequences, and their interaction on moral judgments separately for trials where agents harmed vs. helped (Eq. (3)). Here we observed an effect of harm magnitude on judgments of harmful choices: increases in the number of shocks amplified ascriptions of blame for harmful choices (Study 1, *β*_2−_ = −0.031 ± 0.001, *t* = −21.846, p < 0.001; Study 2, *β*_2−_ = −0.029 ± 0.002, *t* = −12.043, p < 0.001), and decreases in the number of shocks amplified ascriptions of praise for helpful choices (Study 1, *β*_2+_ = 0.027 ± 0.002, *t* = 17.482, p < 0.001; Study 2, *β*_2+_= 0.034 ± 0.003, *t* = 10.384, p < 0.001). Money also exerted an independent effect on judgments. Across both studies, harmful choices were less blameworthy when accompanied by larger profits (Study 1, *β*_3−_ = 0.023 ± 0.001, *t* = 19.850, p < 0.001; Study 2, *β*_3−_ = 0.019 ± 0.002, *t* = 10.608, p < 0.001). Meanwhile, helpful choices were less praiseworthy when they were accompanied by smaller relative to larger costs in Study 1 (*β*_3+_ = −0.055 ± 0.016, *t* = −3.335, p = 0.001). In other words, the presence of incentives mitigated both the condemnation of harmful choices and the praiseworthiness of helpful choices. However, praiseworthiness judgments were not influenced by profit magnitude in Study 2 (*β*_3+_ = 0.023 ± 0.044, *t* = 0.517, p = 0.606). Finally, consistent with the analysis described in Fig. 3a and b and work on the omission bias**,** our linear model showed that harmful actions were judged as more blameworthy than harmful inactions (Study 1, *β*_4−_ = −0.060 ± 0.007, *t* = −8.584, p < 0.001; Study 2, *β*_4−_ = −0.046 ± 0.011, *t* = −4.048, p < 0.001), whereas helpful actions were judged to be more praiseworthy than helpful inactions (Study 1, *β*_4+_ = 0.065 ± 0.008, *t* = 7.888, p < 0.001; Study 2, *β*_4+_ = 0.057 ± 0.016, *t* = 3.543, p < 0.001).

**Fig. 6:**
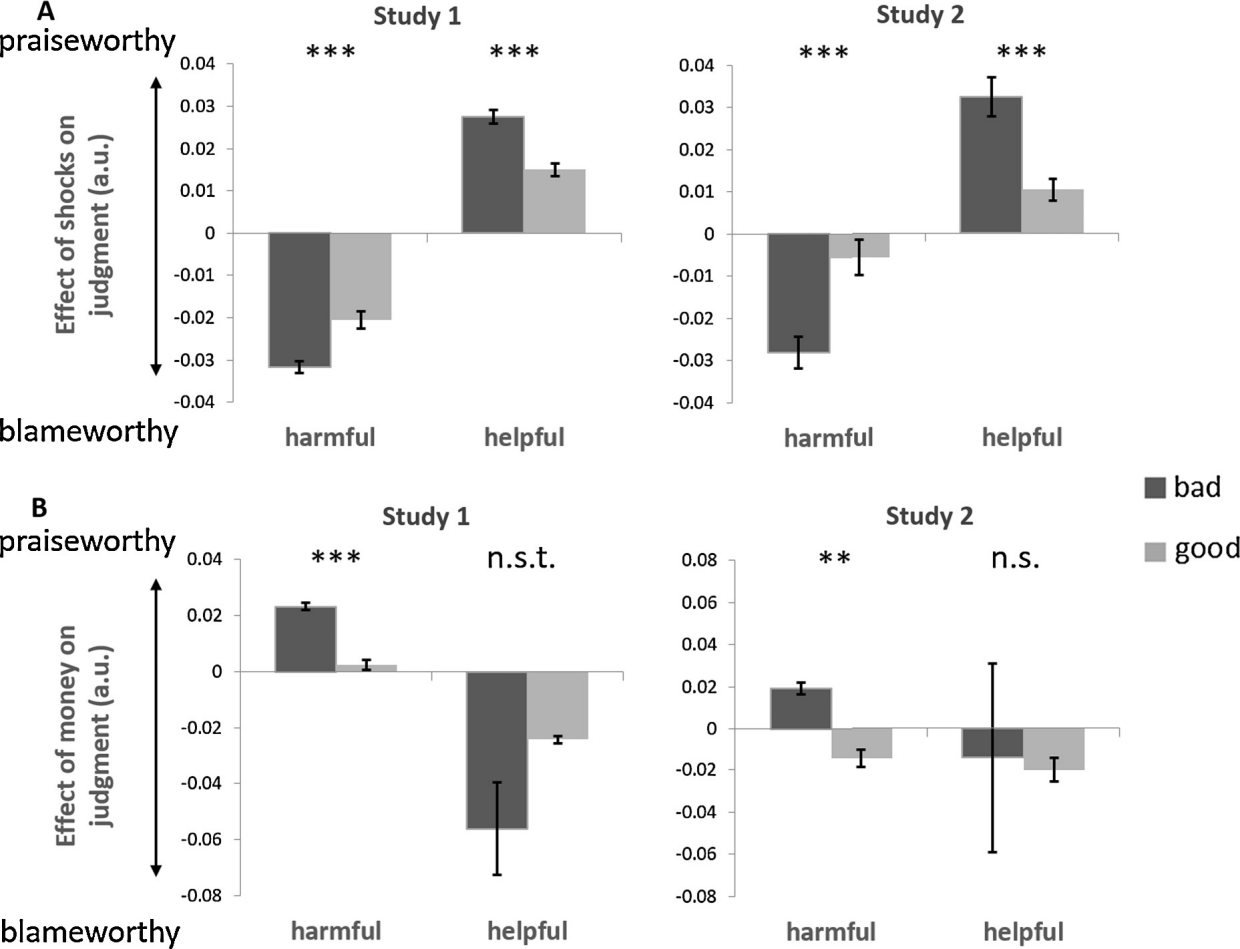
Consequences moderate moral judgments of harmful and helpful actions. Moral judgments were more sensitive to the magnitude of shocks (A) and money (B) for bad agents’ harmful *and* helpful choices, than good agents. Figures illustrate which interactions were statistically significant in Eq. (3). Parameter estimates are displayed on the y-axis A.u. = arbitrary units. ^***^P < 0.001, ^**^P < 0.01; n.s. = not significant, n.s.t. = non-significant trend.

We next investigated the influence of moral character on participants’ sensitivity to consequences for harmful and helpful choices separately. The interaction of character with shocks was significant for both harmful choices (Study 1, *β*_5−_ = 0.011 ± 0.003, *t* = 3.998, p < 0.001; Study 2, *β*_5−_ = 0.023 ± 0.006, *t* = 3.884, p < 0.001) and helpful choices (Study 1, *β*_5+_ = −0.012 ± 0.002, *t* = −5.870, p < 0.001; Study 2, *β*_5+_ = −0.024 ± 0.004, *t* = −5.866, p < 0.001; Fig. 6a). For both harmful and helpful choices, judgments of bad agents were more sensitive to the magnitude of shocks than judgments of good agents. In other words, inferring bad character amplified the effects of increasingly harmful outcomes on blame and also amplified the effects of increasingly helpful outcomes on praise. Character also impacted participants’ sensitivity to money, although these effects were less consistent across harmful and helpful choices. For harmful choices, the magnitude of profit was weighted more strongly in judgments of bad agents than good agents (Study 1, *β*_6−_ = −0.021 ± 0.002, *t* *=* −9.772, p < 0.001; Study 2, *β*_6−_ = −0.033 ± 0.004, *t* *=* −8.646, p < 0.001). In other words, the presence of personal incentives mitigated blameworthiness judgments of harmful choices made by bad agents more strongly than was the case for good agents. However for helpful choices, the magnitude of costs was weighted marginally stronger in judgments of bad agents’ choices than good agents in Study 1 (*β*_6_*_+_* *=* 0.030 ± 0.016, *t* *=* 1.841, p = 0.066), but not Study 2 (*β*_6+_ *=* −0.042 ± 0.044, *t* *=* −0.974, p = 0.330; Fig. 6b).

## 4 Discussion

A person-centered perspective suggests moral judgments encompass evaluation of an agent’s character, in addition to evaluation of choice behavior itself (Uhlmann et al., 2015). In the present study we investigated whether inferences about moral character shape the relative weights placed upon an agent’s consequences and the degree of imputed causation in attributions of blame and praise. To do this we employed a novel approach that involved modelling independent effects of consequences and causation on moral judgments, during observation of decision sequences made by ‘bad’ and ‘good’ agents. Each decision involved a trade-off between personal profit and pain to a victim, and could result from either actions or inactions. By linking agents to a range of harmful and helpful outcomes, that varied in their costs and benefits, we could evaluate how consequences affected judgments of blame and praise (Malle, 2011, Malle et al., 2014). By framing responses as either action or inaction, we could also assess the extent to which an agent’s causal role in bringing about an outcome influenced participants’ blame and praise judgments (Cushman and Young, 2011, Kordes-de Vaal, 1996, Spranca et al., 1991).

We found that inferences about moral character affected the influence of consequences on moral judgments. Consequences were weighted more heavily in judgments of choices made by bad agents, relative to good agents. In other words, the degree of harm and the degree of personal profit resulting from the agent’s choice were more potent factors in blame and praise assessments of bad agents than was the case for good agents. We also found that although judgments were sensitive to whether agents caused the outcomes via an overt action, or via inaction, this factor was not moderated by the character of the agent. That is, causation was similarly weighted when making judgments of good and bad agents’ choices. We note that examining judgments of events caused by actions versus inactions is just one way to study the impact of causal attributions on blame and praise. Other possible approaches include contrasting events caused with physical contact versus no contact, events caused directly versus indirectly, and events caused as a means versus a side-effect (Cushman and Young, 2011, Sloman et al., 2009). Future studies should test the potential importance of character on causation using different manipulations to investigate the generalizability of our findings across multiple manipulations of causation.

In an exploratory analysis, we found that judgments were more sensitive to the magnitude of shocks not only for bad agents’ harmful choices, but also for their helpful choices. Our findings raise a question as to why participant’s praiseworthiness judgments were especially attuned to the helpful consequences of bad agents. Given that bad agents have historically made self-serving decisions, the more intuitive response might be to mitigate sensitivity to the magnitude of helping and consider their apparently ‘altruistic’ behavior as driven by situational factors (e.g., low personal cost to help; Batson and Powell, 2003, Newman and Cain, 2014). From a strict mental state attribution perspective, this finding is perhaps puzzling. However, an important aspect of our experimental design is that no *a priori* information was provided to participants about the morality of the agents. Instead, if participants were motivated to learn about the agents’ moral character, they had to gather information across trials to infer on how averse agents were to harming the victim (i.e., how much each agent was willing to pay to avoid increases in shocks). One possibility is that participants were especially motivated to build accurate predictive models of bad agents, relative to good, because avoiding those who may harm us is an important survival instinct (Cosmides and Tooby, 1992, Johnson et al., 2013). If participants were highly motivated to build a richer model of bad agents, then we would not expect them to neglect relevant information provided in helpful trials. Because people should be particularly motivated to learn about potential social threats, then they should be more attuned to *all* the choices threatening agents make.

Our analysis indicated that harmful choices were less blameworthy when accompanied by larger profits, replicating previous work showing that observers assign less blame to moral violations resulting in large, relative to small, personal benefits (Xie, Yu, Zhou, Sedikides, & Vohs, 2014). Furthermore, this effect was more pronounced for bad agents than good agents. That is, the presence of personal incentives (i.e., money) mitigated blameworthiness judgments of harmful choices made by bad agents more strongly than was the case for good agents. Meanwhile, we obtained less consistent findings for the effect of personal incentives on judgments of helpful choices across Studies 1 and 2. First, the presence of incentives mitigated the praiseworthiness of helpful choices in Study 1, but not Study 2. Second, judgments of bad agents’ choices were marginally more sensitive to the magnitude of incentives for helpful choices in Study 1, but not Study 2. Thus, it is possible that character only moderates the effect of personal incentives on the blameworthiness of harmful choices, and not the praiseworthiness of helpful choices. However, we caution that the range in the magnitude of incentives for helpful choices was very small for bad agents (as the maximum amount of money bad agents would give up to help was £1.00; Supplementary Table S3). Furthermore, other work has shown that agents who help others, in the absence of personal incentives, are judged more favorably than those whose helpful choices can be explained by incentives (Newman & Cain, 2014). Thus, an alternative possibility is that the range in money for helpful choices was too small to observe (a) a main effect of money for helping in Study 2, and (b) an interaction between character and money for helping.

Another limitation of our experimental design is that consequences were not dissociated from the intentions of the agents. Thus, it is unclear whether greater sensitivity to consequences for bad, relative to good, agents is driven by an increased sensitivity to intent or consequences. Future studies could dissociate intent and consequences using the current experimental design by randomly varying whether the agents’ intentions are actually implemented. We might speculate that the findings here are motivated by consequences rather than intentions in light of recent work on how people blame and punish accidents, which dissociate consequences and intent (Cushman et al., 2009). Research on judging accidents shows that moral judgments are sensitive to both consequences and intent (Ahram et al., 2015, Cushman, 2008, Cushman et al., 2009, Martin and Cushman, 2015, Oswald et al., 2005), but consequences may play a more dominant role when judging accidents (Cushman et al., 2009). Notably, sensitivity to accidental consequences appear to matter significantly more when people are asked how much blame or punishments should be attributed to the behavior, than when asked how wrong or permissible it was (Cushman, 2008). Martin and Cushman explain this finding by arguing that punitive behaviors signal to others to adjust their actions (Martin and Cushman, 2015, Martin and Cushman, 2016). In this sense, punishment is adaptive to the extent that it improves one’s own chance of engaging in future cooperation with past wrongdoers, and thus serves as a ‘teaching signal’. If punishment and reward serve as teaching signals, we might expect them to be more readily endorsed as a function of outcome severity when we infer bad character. That is, teaching signals should be preferentially directed towards those who need to be taught. While we do need to teach someone with a history of bad behavior right from wrong, this is less necessary when we consider someone who has already learned how to cooperate.

## 5 Conclusion

We employed novel methods to investigate the effects of moral character on how people integrate information about consequences and causation in judgments of choices to help or harm a victim. We validated these methods by replicating previous findings that the magnitude of consequences and causation shape attributions of blame for harmful choices and praise for helpful choices. Character moderated the effects of consequences on judgments, with consequences weighting more strongly in judgments of bad relative to good agents. Our findings support a person-centered approach to moral judgment, and suggest avenues for future research investigating how impressions of morality are formed over time and how these evolving impressions shape subsequent moral judgments.
