# Supplementary material for: Inferences about moral character moderate the impact of consequences on blame and praise
Source: Cognition. 2017 Oct;167:201–11. doi: 10.1016/j.cognition.2017.05.004 (PMC5552615; doi:10.1016/j.cognition.2017.05.004)
Supplement: Supplementary data 1 [file mmc1.docx]

**Supplemental Materials**

**Moral Judgment Task Instructions**

Participants assigned the role of the receiver were instructed in both verbal and written form as follows:

“You have been randomly assigned to the role of **Receiver.** The other participant in this session has been randomly assigned to the role of **Decider.**

In this study, the Decider makes a series of decisions. Each decision involves choosing between a smaller amount of money plus a smaller number of shocks, or a larger amount of money plus a larger number of shocks. The Decider receives the money, while you (as the Receiver) receive the shocks.

The shock intensity level is always set to **level 8** – i.e., just below the “intolerable” pain level.

At the beginning of each decision, a certain number of shocks will be allocated. Next, a new number of shocks will appear. The Decider must decide whether to switch to the new number of shocks, or to keep it the same.

The Decider always has two options:

- - Do nothing, and receive £10
  - Switch to the new number of shocks and receive a different amount of money

The amount of money the Decider receives from switching to the new number of shocks will be indicated at the time he/she makes his/her decision.”

Next, subjects were previewed to the screen design for the moral economic exchange task and verbally instructed simultaneously.


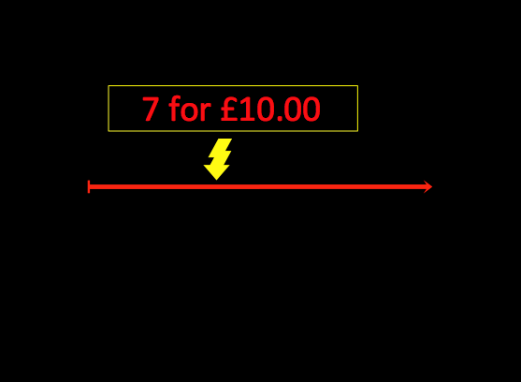


“First the decider will see a default number of shocks for you, and the payment they would receiver in they did nothing.


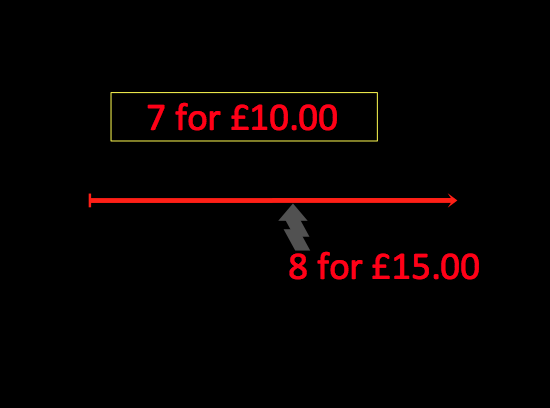


Next, they see an alternative option, which is a different number of shocks for you and a different amount of money. The new amount can be either higher or lower than the default.


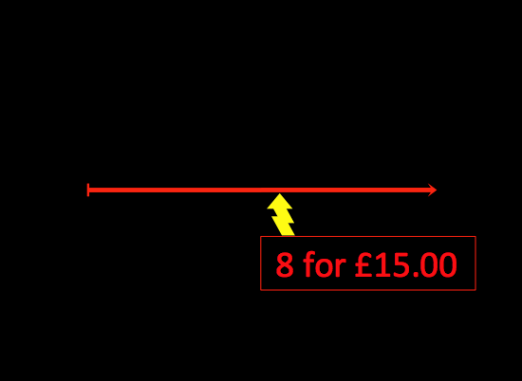


If the decider chooses to switch to the alternative option, they must press a button on the keyboard, at which point, the chosen option is highlighted.


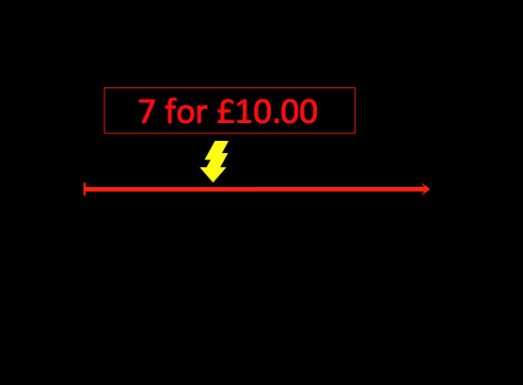


However, if the decider chooses to stay with the default, then they do nothing and the default will become highlighted with a red box.

Throughout the study the Decider will make a series of decisions like these (approximately 160). However, no shocks will be delivered during the decision-making task.

Instead, at the end of the task, **one trial** will be randomly selected, and the Decider’s choice from that trial will be actually implemented. There is a chance that you might not receive any shocks. However, if the Decider’s choice results in a positive number of shocks for you, then you will receive these shocks at the end of the study.

While the real Decider is making his/her decisions next door, you will make **moral judgments** about four other Deciders, whose decisions we will present to you.

You will judge each of these Deciders one at a time. We will show you a series of decisions made by each Decider. After each decision, we would like you to judge how morally right or wrong was the Decider’s choice.

To indicate your judgment, we would like you to respond on a scale ranging from *blameworthy* to *praiseworthy.* If you think the choice was extremely nasty and deserves a lot of blame, you should respond at the extreme left side of the scale. If you think the choice was extremely admirable and deserves a lot of praise, you should respond at the extreme right of the scale. If you think the choice was neither blameworthy nor praiseworthy, respond in the middle of the scale.

After you observe each choice, you will use the mouse to indicate your judgment of the choice.”

Subjects were then previewed to the rating screen and instructed how to use the rating scale.

“You will judge a series of 30-32 decisions for each of four Deciders.

Remember, on each trial you will see:

- - The initial number of shocks
  - The new number of shocks
  - The money the Decider receives for switching to the new number of shocks
  - The Decider’s choice

You will then use the mouse to indicate your judgment of the Decider’s choice.

After you have rated all the decisions for a given Decider, we will ask you a few questions about your general impressions of this person.”

**Creating trials and simulating choices**

*Agents B1 and G1*: A set of 15 “action” trials was produced with one trial at each indifference point evenly spaced between ln(κ) = -4 to ln(κ) = 2. We created this stimulus set by randomly generating different pairs of Δs and Δm, and computing the indifference point each pair was closest to. This process was repeated until a pair of Δs and Δm was found for each indifference point. We then created a set of 15 matched “inaction” trials by swapping the values of the default option and alternative option, to produce a full set of 30. We then simulated the decisions that each agent would make based on their personal κ [ln(κ) agent 1 = -2, ln(κ) agent 3 = 0].

*Agents B2 and G2*: A set of 16 “action” trials was produced by randomly generating Δs between 1 and 9, and choosing a value for Δm that matched the value difference (Vact) between agents. Vact is the difference in utility between the two option, and is computed using:

Vact = Δm – Δs*exp[ln(κ_i_)]

Where κ_i_ is the κ for agent i. For each trial we fixed Vact to a randomly determined value between -1 and 1. Because Δs was equal for both agents, we solved for Δm by substituting individual κ values into the value difference equation:

Δm = Vact + Δs*exp[ln(κ_i_)]

We then created a set of 16 matched “inaction” trials by swapping the values of the default option and alternative option, to produce a full set of 32.

The change in shocks and money for harmful trials (and helpful trials) were sufficiently decorrelated (<0.7) to enable us to investigate independent effects of shocks and money in our parametric analyses (Dormann et al. 2013). Across all agents, the correlation between Δm and Δs for harmful trials was equal to 0.124 and the correlation between Δm and Δs for helpful trials was equal to 0.285 (Table S1).

Three sequences of trials were generated and randomized across participants using the above methods.

**Table S1:** Correlations between money and shock regressors in Eq. 2

|  | Script 1 | Script 2 | Script 3 |
| --- | --- | --- | --- |
| All agents | -0.276 | -.358 | -.241 |
| Bad agents | -0.455 | -.435 | -.405 |
| Good agents | -0.249 | -.468 | -.296 |

The correlations between money and shock regressors in Eq. 2 were not significantly different for bad and good agents in Script 1 (Z = -1.29, p = 0.19), Script 2 (Z = 0.23, p = 0.82) or Script 3 (Z = -0.68, p = 0.50).

**Table S2:** Full Trial Set for an exemplary trial sequence. We highlight trials where agents B1 and G1 make identical choices.

Agent B1:

| **default shocks** | **default money** | **alternative shocks** | **alternative money** | **switch** |
| --- | --- | --- | --- | --- |
| 13 | 10 | 19 | 10.1 | no |
| 12 | 10 | 14 | 11.7 | yes |
| 6 | 10 | 8 | 10.7 | yes |
| 1 | 10 | 0 | 2.5 | no |
| 8 | 10 | 0 | 8 | no |
| 7 | 10 | 2 | 9.5 | yes |
| 20 | 10 | 12 | 5.8 | no |
| 4 | 10 | 6 | 16.1 | yes |
| 4 | 10 | 11 | 19.6 | yes |
| 6 | 10 | 4 | 3.9 | no |
| 19 | 10 | 13 | 9.9 | yes |
| 16 | 10 | 17 | 14.9 | yes |
| 9 | 10 | 16 | 10.2 | no |
| 8 | 10 | 6 | 9.3 | no |
| 15 | 10 | 20 | 10.8 | yes |
| 0 | 10 | 8 | 12 | yes |
| 9 | 10 | 12 | 10.2 | no |
| 16 | 10 | 9 | 9.8 | yes |
| 10 | 10 | 19 | 10.4 | no |
| 11 | 10 | 4 | 0.4 | no |
| 1 | 10 | 5 | 18.6 | yes |
| 19 | 10 | 10 | 9.6 | yes |
| 20 | 10 | 15 | 9.2 | no |
| 5 | 10 | 1 | 1.4 | no |
| 12 | 10 | 9 | 9.8 | yes |
| 12 | 10 | 20 | 14.2 | yes |
| 17 | 10 | 16 | 5.1 | no |
| 14 | 10 | 12 | 8.3 | no |
| 0 | 10 | 1 | 17.5 | yes |
| 2 | 10 | 7 | 10.5 | no |

Agent B2:

| **default shocks** | **default money** | **alternative shocks** | **alternative money** | **switch** |
| --- | --- | --- | --- | --- |
| 19 | 10 | 12 | 8.8 | no |
| 20 | 10 | 16 | 9.2 | no |
| 20 | 10 | 11 | 9.5 | yes |
| 14 | 10 | 5 | 8.1 | no |
| 11 | 10 | 3 | 8.8 | no |
| 3 | 10 | 0 | 9.9 | yes |
| 17 | 10 | 16 | 9.4 | no |
| 9 | 10 | 6 | 9.8 | yes |
| 0 | 10 | 3 | 10.1 | no |
| 5 | 10 | 14 | 11.9 | yes |
| 0 | 10 | 8 | 10.1 | no |
| 4 | 10 | 12 | 10.9 | no |
| 12 | 10 | 19 | 11.2 | yes |
| 14 | 10 | 16 | 10.2 | no |
| 16 | 10 | 11 | 9.7 | yes |
| 11 | 10 | 20 | 10.5 | no |
| 10 | 10 | 12 | 10.5 | yes |
| 16 | 10 | 14 | 9.8 | yes |
| 10 | 10 | 4 | 9.5 | yes |
| 6 | 10 | 9 | 10.2 | no |
| 5 | 10 | 9 | 11.5 | yes |
| 11 | 10 | 16 | 10.3 | no |
| 8 | 10 | 0 | 9.9 | yes |
| 9 | 10 | 5 | 8.5 | no |
| 3 | 10 | 11 | 11.2 | yes |
| 16 | 10 | 17 | 10.6 | yes |
| 6 | 10 | 2 | 9.3 | no |
| 16 | 10 | 20 | 10.8 | yes |
| 12 | 10 | 4 | 9.1 | yes |
| 12 | 10 | 10 | 9.5 | no |
| 2 | 10 | 6 | 10.7 | yes |
| 4 | 10 | 10 | 10.5 | no |

Agent G1:

| **default shocks** | **default money** | **alternative shocks** | **alternative money** | **switch** |
| --- | --- | --- | --- | --- |
| 16 | 10 | 9 | 9.8 | yes |
| 9 | 10 | 12 | 10.2 | no |
| 17 | 10 | 16 | 5.1 | no |
| 12 | 10 | 20 | 14.2 | no |
| 8 | 10 | 6 | 9.3 | yes |
| 19 | 10 | 10 | 9.6 | yes |
| 2 | 10 | 7 | 10.5 | no |
| 1 | 10 | 5 | 18.6 | yes |
| 0 | 10 | 1 | 17.5 | yes |
| 19 | 10 | 13 | 9.9 | yes |
| 9 | 10 | 16 | 10.2 | no |
| 15 | 10 | 20 | 10.8 | no |
| 0 | 10 | 8 | 12 | no |
| 20 | 10 | 15 | 9.2 | yes |
| 14 | 10 | 12 | 8.3 | yes |
| 10 | 10 | 19 | 10.4 | no |
| 20 | 10 | 12 | 5.8 | yes |
| 13 | 10 | 19 | 10.1 | no |
| 1 | 10 | 0 | 2.5 | no |
| 7 | 10 | 2 | 9.5 | yes |
| 8 | 10 | 0 | 8 | yes |
| 4 | 10 | 6 | 16.1 | yes |
| 12 | 10 | 14 | 11.7 | no |
| 16 | 10 | 17 | 14.9 | yes |
| 11 | 10 | 4 | 0.4 | no |
| 12 | 10 | 9 | 9.8 | yes |
| 4 | 10 | 11 | 19.6 | yes |
| 6 | 10 | 8 | 10.7 | no |
| 5 | 10 | 1 | 1.4 | no |
| 6 | 10 | 4 | 3.9 | no |

Agent G2:

| **default shocks** | **default money** | **alternative shocks** | **alternative money** | **switch** |
| --- | --- | --- | --- | --- |
| 8 | 10 | 0 | 3 | yes |
| 5 | 10 | 14 | 19.7 | yes |
| 0 | 10 | 3 | 12.7 | no |
| 9 | 10 | 6 | 7.2 | yes |
| 4 | 10 | 12 | 17.8 | no |
| 5 | 10 | 9 | 15 | yes |
| 17 | 10 | 16 | 8.6 | no |
| 14 | 10 | 5 | 0.3 | no |
| 16 | 10 | 17 | 11.4 | yes |
| 19 | 10 | 12 | 2.7 | no |
| 20 | 10 | 11 | 1.7 | yes |
| 16 | 10 | 14 | 8.1 | yes |
| 2 | 10 | 6 | 14.2 | yes |
| 11 | 10 | 20 | 18.3 | no |
| 11 | 10 | 3 | 1.9 | no |
| 10 | 10 | 12 | 12.2 | yes |
| 3 | 10 | 0 | 7.3 | yes |
| 12 | 10 | 4 | 2.2 | yes |
| 20 | 10 | 16 | 5.7 | no |
| 11 | 10 | 16 | 14.6 | no |
| 9 | 10 | 5 | 5 | no |
| 10 | 10 | 4 | 4.3 | yes |
| 0 | 10 | 8 | 17 | no |
| 3 | 10 | 11 | 18.1 | yes |
| 12 | 10 | 10 | 7.8 | no |
| 16 | 10 | 11 | 5.4 | yes |
| 16 | 10 | 20 | 14.3 | For trial etyes |
| 14 | 10 | 16 | 11.9 | no |
| 12 | 10 | 19 | 17.3 | yes |
| 4 | 10 | 10 | 15.7 | no |
| 6 | 10 | 2 | 5.8 | no |
| 6 | 10 | 9 | 12.8 | no |

|  | **Bad Agents** | | **Good Agents** | |
| --- | --- | --- | --- | --- |
|  | *B1* | *B2* | *G1* | *G2* |
| **shocks range** | 17.0 | 18.0 | 16.0 | 18.0 |
| **money range** | 10.1 | 2.8 | 13.8 | 18.0 |
| **shocks variance** | 5.4 | 6.0 | 4.7 | 5.9 |
| **money variance** | 3.5 | 0.8 | 4.3 | 5.8 |

**Table S3:** Range and Variance in shocks and money for each agent in an exemplary trial sequence.

**Replacing categorical objective ‘character’ regressor with subjective kindness ratings:**

For the main analyses we analyzed the data using a model that included an objective categorical regressor describing the independent effect of *good* agents on moral judgment. An alternative approach is to replace this objective categorical regressor with participants’ subjective ratings that were collected at the end of the task as a manipulation check. Modelling participant’s responses in this way yielded comparable results to those reported in the paper. Table S4a and S4b presents the full results from this analysis for Study 1 and 2, respectively, using equation 2 from the main text. Table S4c and S4d presents the full results from this analysis for Study 1 and 2, respectively, using equation 3 from the main text.

**Table S4a:**

Study 1:

|  | Estimate | SEM | *t*-statistic | p-value |
| --- | --- | --- | --- | --- |
| β1 | 0.178 | 0.012 | 15.330 | <0.001 |
| β2 | 0.016 | 0.002 | 8.324 | <0.001 |
| β3 | 0.030 | 0.002 | 17.268 | <0.001 |
| β4 | 0.128 | 0.011 | 11.815 | <0.001 |
| β5 | -0.003 | 0.003 | -0.931 | 0.352 |
| β6 | -0.016 | 0.003 | -5.767 | <0.001 |
| β7 | -0.006 | 0.018 | -0.367 | 0.714 |
| c | 0.343 | 0.011 | 31.751 | <0.001 |

**Table S4b:**

Study 2:

|  | Estimate | SEM | *t*-statistic | p-value |
| --- | --- | --- | --- | --- |
| β1 | 0.161 | 0.031 | 5.234 | 0.000 |
| β2 | 0.022 | 0.004 | 5.175 | 0.000 |
| β3 | 0.026 | 0.004 | 6.700 | 0.000 |
| β4 | 0.120 | 0.025 | 4.781 | 0.000 |
| β5 | -0.012 | 0.007 | -1.867 | 0.062 |
| β6 | -0.015 | 0.007 | -2.220 | 0.027 |
| β7 | -0.019 | 0.038 | -0.484 | 0.628 |
| c | 0.401 | 0.024 | 16.783 | 0.000 |

**Table S4c:**

Study 1:

|  | Estimate | SEM | *t*-statistic | p-value |
| --- | --- | --- | --- | --- |
| β1 | 0.226 | 0.019 | 12.015 | 0.000 |
| β2- | -0.033 | 0.002 | -14.276 | 0.000 |
| β2+ | 0.030 | 0.002 | 15.297 | 0.000 |
| β3- | 0.028 | 0.002 | 14.351 | 0.000 |
| β3+ | -0.025 | 0.004 | -6.138 | 0.000 |
| β4- | -0.061 | 0.005 | -11.619 | 0.000 |
| β4+ | 0.077 | 0.005 | 14.664 | 0.000 |
| β5- | 0.007 | 0.004 | 1.659 | 0.097 |
| β5+ | -0.012 | 0.003 | -3.678 | 0.000 |
| β6- | -0.023 | 0.004 | -6.645 | 0.000 |
| β6+ | 0.005 | 0.006 | 0.888 | 0.375 |
| c | 0.327 | 0.014 | 22.708 | 0.000 |

**Table S4d:**

Study 2:

|  | Estimate | SEM | *t*-statistic | p-value |
| --- | --- | --- | --- | --- |
| β1 | 0.287 | 0.043 | 6.668 | 0.000 |
| β2- | -0.030 | 0.005 | -5.978 | 0.000 |
| β2+ | 0.041 | 0.005 | 8.811 | 0.000 |
| β3- | 0.024 | 0.004 | 6.325 | 0.000 |
| β3+ | -0.048 | 0.020 | -2.348 | 0.019 |
| β4- | -0.048 | 0.009 | -5.175 | 0.000 |
| β4+ | 0.041 | 0.009 | 4.370 | 0.000 |
| β5- | 0.002 | 0.009 | 0.250 | 0.802 |
| β5+ | -0.032 | 0.007 | -4.395 | 0.000 |
| β6- | -0.024 | 0.006 | -3.785 | 0.000 |
| β6+ | 0.031 | 0.027 | 1.148 | 0.251 |
| c | 0.345 | 0.032 | 10.818 | 0.000 |

β_1_ : weight on kindness rating

β_2_ : weight on shock magnitude

β_3_ : weight on profit magnitude

β_4-_: weight on causation

β_5_ : weight on shock magnitude*character interaction

β_6_ : weight on profit magnitude*character interaction

β_7_ : weight on causation*character interaction

*c*: intercept

- : harmful choices

+ : helpful choices

**Standardized regression coefficients**

We performed our analyses with standardized regression coefficients (which converts all parameter values into a standard space using z-scores) and we find the same general pattern of results reported using unstandardized regression coefficients. . Table S5a and S5b presents the full results from this analysis for Study 1 and 2, respectively, using equation 2 from the main text. Table S5c and S5d presents the full results from this analysis for Study 1 and 2, respectively, using equation 3 from the main text.

**Table S5: parameter estimates using standardized regression coefficients**

**Table S5a:**

Study 1:

|  | Estimate | SEM | *t*-statistic | p-value |
| --- | --- | --- | --- | --- |
| β1 | 0.040 | 0.004 | 8.974 | <0.001 |
| β2 | 0.088 | 0.007 | 13.240 | <0.001 |
| β3 | 0.087 | 0.004 | 23.707 | <0.001 |
| β4 | 0.127 | 0.007 | 17.708 | <0.001 |
| β5 | -0.024 | 0.009 | -2.535 | 0.011 |
| β6 | -0.032 | 0.005 | -6.787 | <0.001 |
| β7 | 0.002 | 0.010 | 0.177 | 0.860 |
| c | 0.505 | 0.011 | 47.284 | <0.001 |

**Table S5b:**

Study 2:

|  | Estimate | SEM | *t*-statistic | p-value |
| --- | --- | --- | --- | --- |
| β1 | 0.033 | 0.008 | 4.144 | <0.001 |
| β2 | 0.099 | 0.011 | 8.815 | <0.001 |
| β3 | 0.075 | 0.006 | 12.333 | <0.001 |
| β4 | 0.097 | 0.013 | 7.373 | <0.001 |
| β5 | -0.052 | 0.016 | -3.166 | 0.002 |
| β6 | -0.049 | 0.012 | -4.214 | <0.001 |
| β7 | 0.008 | 0.018 | 0.446 | 0.656 |
| c | 0.557 | 0.019 | 29.817 | <0.001 |

**Table S5c:**

Study 1:

|  | Estimate | SEM | *t*-statistic | p-value |
| --- | --- | --- | --- | --- |
| β1 | 0.033 | 0.015 | 2.258 | 0.024 |
| β2- | -0.080 | 0.004 | -21.846 | <0.001 |
| β2+ | 0.094 | 0.005 | 17.482 | <0.001 |
| β3- | 0.071 | 0.004 | 19.850 | <0.001 |
| β3+ | -0.118 | 0.035 | -3.335 | 0.001 |
| β4- | -0.060 | 0.005 | -11.251 | <0.001 |
| β4+ | 0.076 | 0.005 | 14.273 | <0.001 |
| β5- | 0.027 | 0.007 | 3.998 | <0.001 |
| β5+ | -0.043 | 0.007 | -5.870 | <0.001 |
| β6- | -0.064 | 0.007 | -9.772 | <0.001 |
| β6+ | 0.065 | 0.036 | 1.841 | 0.066 |
| c | 0.518 | 0.018 | 29.279 | <0.001 |

**Table S5d:**

Study 2:

|  | Estimate | SEM | *t*-statistic | p-value |
| --- | --- | --- | --- | --- |
| β1 | 0.072 | 0.015 | 4.742 | <0.001 |
| β2- | -0.068 | 0.006 | -12.043 | <0.001 |
| β2+ | 0.113 | 0.011 | 10.384 | <0.001 |
| β3- | 0.066 | 0.006 | 10.608 | <0.001 |
| β3+ | 0.021 | 0.040 | 0.517 | 0.606 |
| β4- | -0.041 | 0.009 | -4.422 | <0.001 |
| β4+ | 0.034 | 0.009 | 3.615 | <0.001 |
| β5- | 0.053 | 0.014 | 3.884 | <0.001 |
| β5+ | -0.079 | 0.013 | -5.866 | <0.001 |
| β6- | -0.113 | 0.013 | -8.646 | <0.001 |
| β6+ | -0.039 | 0.040 | -0.974 | 0.330 |
| c | 0.539 | 0.023 | 23.683 | <0.001 |

β_1_ : weight on good agent

β_2_ : weight on shock magnitude

β_3_ : weight on profit magnitude

β_4-_: weight on causation

β_5_ : weight on shock magnitude*character interaction

β_6_ : weight on profit magnitude*character interaction

β_7_ : weight on causation*character interaction

*c*: intercept

- : harmful choices

+ : helpful choices
